# Designing a Peer Navigation Program Alongside Women With Lived Experience of Domestic, Family, and Sexual Violence

**DOI:** 10.1111/hex.70877

**Published:** 2026-09-22

**Authors:** Zoe King, Heshani Samantha De Silva, Katie Alexander, Liz John, Shiana Harris, Teresa, Renee Fiolet, Sally Connell, Sally Nathan, Ruth Wells, Kelsey Hegarty, Patricia Cullen

**Affiliations:** ^1^ School of Population Health, University of New South Wales Sydney New South Wales Australia; ^2^ Department of Social Work The University of Melbourne Melbourne Victoria Australia; ^3^ Safer Families Centre of Research Excellence, University of Melbourne Melbourne Victoria Australia; ^4^ Centre for Quality and Patient Safety Research, Institute for Health Transformation, Deakin University Melbourne Victoria Australia; ^5^ Department of General Practice and Primary Care The University of Melbourne Melbourne Victoria Australia; ^6^ Independent Consultant Shellharbour New South Wales Australia; ^7^ Discipline of Psychiatry and Mental Health, School of Clinical Medicine, University of New South Wales Sydney New South Wales Australia; ^8^ The Royal Women's Hospital Parkville Victoria Australia

**Keywords:** community health workers, community participation, domestic violence, intimate partner violence, patient navigation, primary health care

## Abstract

**Background:**

Domestic, family, and sexual violence (DFSV) is a significant public health issue. There are currently large gaps in prevention, early intervention, and longer‐term recovery and healing support for victim‐survivors of DFSV. Implementing peer navigation programs to support victim‐survivors has the potential to address some of these gaps. Yet there is a lack of current research on the components necessary to implement these programs, including in primary care settings.

**Objective:**

To develop core components of a DFSV peer navigation program tailored to primary care settings.

**Methods:**

A participatory design approach bringing together women with lived experience of DFSV, health practitioners, and researchers.

**Results:**

We designed five core components of a peer navigation program: (1) Walking Alongside: Peer‐Informed Relational Care; (2) Peer Navigation as Practical Support; (3) Safe and Ethical Practice in Peer Navigation; (4) Role Clarity and Boundary‐Setting; and (5) Organisational Supports for Peer Navigation Practice. In presenting these results, we also highlight reflections on our design process.

**Conclusion:**

The implementation of peer navigation programs for victim‐survivors can provide survivor‐centred relational and practical support. Ensuring effective implementation of peer navigation programs must address key challenges, particularly in ensuring safety and wellbeing of victim‐survivors and peer navigators through DFSV‐informed primary care practice. These findings can inform the implementation of peer navigation programs for victim‐survivors to promote care that prioritises trusting relationships, agency, and pragmatic support.

**Patient or Public Contribution:**

This research centred the lived expertise of victim‐survivors. We established a Working Group of women with lived experience of DFSV, health practitioners, and researchers to design the components of a peer navigation program, using our lived, living, and learned (professional) expertise. Women with lived experience were involved in the study design, as members of the Working Group, and in manuscript authorship.

## 1 Introduction

Domestic, family and sexual violence (DFSV) describes multiple forms of violence including violence perpetrated by a partner, violence perpetrated within families, and sexual violence [1]. It is estimated that one in three women globally have experienced intimate partner and/or sexual violence [2]. Victim‐survivors of DFSV experience immediate, short, and long‐term health impacts including a range of physical and mental health outcomes such as traumatic stress, pain, complex trauma, emotional distress, and suicidality [3, 4]. In this paper we use the term “victim‐survivors” to describe people who have experienced DFSV, although we acknowledge not all people who have experienced DFSV identify as “victims” and/or “survivors”. For those who experience multiple forms of violence (psychological, sexual, physical violence, etc.), the impacts can be cumulative, with increased likelihood of poorer health outcomes and increased vulnerability to health risks, particularly suicidality [5]. Given the contribution to burden of disease and the potentially lifelong health consequences for individuals, families, and communities, DFSV represents a significant public health issue.

### 1.1 Role of Health Services in Responding to DFSV

Health services have a vital role in preventing and responding to DFSV through resourcing, shaping policy, and building workforce capacity in services such as primary care, emergency departments, mental and community health services. Health services are a commonly used source of support for victim‐survivors [6, 7], with some research indicating that health services are the most frequently used formal support system, more so than DFSV specialist services, police or legal supports [8, 9, 10].

Despite the importance of prevention and response efforts, health services and the workforce encounter substantial barriers in providing safe and effective care for victim‐survivors [6]. A qualitative meta‐synthesis reported that health practitioners identify structural barriers to providing high quality care to victim‐survivors, including short appointment times that limited the development of rapport, feeling unprepared to support victim‐survivors, including not knowing where to refer, and lack of policies and procedures at the systems‐level [11].

From victim‐survivors' perspectives, shortcomings in care are well documented [12, 13, 14]. Victim‐survivors report some health practitioners not providing trauma‐informed care, including misidentification of DFSV, lack of follow‐up after disclosures, and practitioners pathologising DFSV [8]. Victim‐survivors also describe complexity in navigating siloed services, lack of culturally safe services, and experiences of racism, misogyny, bullying, and discrimination [12, 13, 14]. These issues can undermine provision of care and harm all victim‐survivors, particularly those who are already disproportionately harmed by DFSV, including First Nations communities, culturally and linguistically diverse communities, LGBTQ+ people, people with disabilities, and rural and remote communities [15, 16, 17, 18, 19].

By contrast, victim‐survivors have identified that services providing emotional and practical support are key to overcoming some of these challenges [8, 20, 21, 22]. A qualitative meta‐synthesis of experiences disclosing DFSV to health practitioners found that some victim‐survivors highly valued emotional connection with health practitioners [20]. At the same time, victim‐survivors want health practitioners to “do more than just listen” and provide practical support, such as assistance with insurance and connection to community services [20, p. 18]. Yet the persistence of structural barriers identified by both the workforce and victim‐survivors limit provision of this support, impeding safe and effective care.

### 1.2 Improving Responses to DFSV Through Peer Work

Peer work, where those with lived experience of DFSV provide support and work alongside clinicians, is a promising approach to address some of these challenges and better support victim‐survivors [23, 24, 25]. Peer workers are people who are trained and supported to use their lived experience to offer genuine connection, empathy and practical support to others navigating recovery and healing [23, 26, 27, 28, 29]. Widespread adoption of peer work in mental health, and other healthcare settings, has seen the professionalisation and growth of peer worker roles, including peer navigators [27, 28]. Peer navigators are a type of peer worker who help people ‘navigate’ complex health and social systems, by providing practical support to access services and overcome barriers to care, in addition to providing peer support [30, 31, 32, 33, 34].

Victim‐survivors have long provided informal or volunteer‐based peer support to each other, including one‐on‐one support and peer support groups that are in‐person, through community organisations, and online forums. In terms of formalised one‐on‐one peer work or navigation, there is limited research [23]. To our knowledge, there are few studies exploring peer navigation programs for victim‐survivors [35, 36, 37], and none based in primary care settings. In this study, we use participatory design methods to develop core components of a DFSV peer navigation program in primary care settings.

## 2 Materials and Methods

### 2.1 Study Design

We use a participatory design approach, bringing together women with lived experience of DFSV, health practitioners and researchers to explore key considerations and collaborate on designing core components of a DFSV peer navigation program in primary care [38, 39, 40]. A participatory design approach actively partners with service ‘users’ who are experts by their own lived experience and apply this expertise in the design process [38]. Given there is often heterogeneity regarding the reporting of participatory methods, we use a systematic review of design workshops for health information technologies that provides recommendations for reporting [38, 41]. This study was approved by the University of New South Wales Sydney Human Research Ethics Committee (iRECS7467).

### 2.2 Study Context

This work informs a forthcoming multicomponent intervention, which includes the provision of peer navigation for victim‐survivors presenting to Australian primary care clinics. In the Australian health system, primary care clinics are central, with family doctors (general practitioners) typically providing a first point of contact and coordinating access to other services [42, 43].

### 2.3 Recruitment

Our group of designers (hereafter “Working Group”) all had prior co‐research and/or service design experience. Five women with lived experience of DFSV were recruited through the WEAVERs, a network run by the University of Melbourne's Safer Families Centre that embeds lived experience of DFSV into research and training [44]. The Safer Families Centre posted the co‐research opportunity in their newsletter and following expressions of interest, five WEAVERs were sent study information. All five accepted the invitation and consented to the study.

Health practitioners were recruited through the networks of researchers for their expertise in DFSV, social work, primary care, and service design. Three health practitioners and a researcher with expertise in the field were invited to join and provided with study information. Two health practitioners accepted and consented to the study; the others declined due to workload and scheduling constraints.

The Working Group included five women from the WEAVERs network (hereafter “WEAVERs”), two health practitioners from social work and nursing professions, and two public health researchers (ZK, PC) who have experience in qualitative research and co‐design and clinical experience as a psychologist (PC). While ZK and PC took on practical roles of coordination and facilitation, they were also active members of the Working Group, engaged in shared priority setting, collaborative planning, and design.

### 2.4 Positionality

Recruitment was purposive, aiming to include diversity in lived, living, and learned (professional) expertise [45]; however, in practice, these forms of expertise often intersected, with designers drawing on multiple sources of knowledge. For example, some WEAVERs brought their professional experiences in health, community, and government, while some health practitioners and researchers brought their lived experience of DFSV. Some members shared aspects of their identity throughout the workshops—neurodiversity, cultural background—and how this shaped their contributions to program design. For those who drew on their social or cultural identities, they were mindful that they contributed from their own standpoints rather than as representative of whole communities.

### 2.5 Design Process

#### 2.5.1 Informal Session and Priority Setting

Prior to beginning the first workshop, the WEAVERs and researchers met over Zoom for an informal relationship‐building session. We discussed how we wanted to work together, including processes for determining design priorities. The WEAVERs determined ZK and PC would draw on a forthcoming systematic review on trauma‐and‐violence‐informed peer navigation to create a tentative structure for the workshops (Table 1, labelled a–e). Throughout the process, the Working Group iteratively refined these priorities enabling ZK and PC to tailor activities for subsequent workshops.

**Table 1:** Processes and activities of design workshops.

Workshop	Overview	Activities
Informal relationship‐building session 1‐h meeting, 1‐h reading time; *n* = 7 (WEAVERs, ZK and PC only)	Introductions and getting to know each other	Introductions and reflections on our hopes for, and interest in, joining the Working Group.
	Introduction to the project and answering questions	Short slide deck to provide visuals during project introduction; whole group discussion on the purpose of the design workshops, along with reflections on trauma‐and‐violence‐informed group processes and insights into what has worked well in prior co‐research.
	Project guide	Project guide was shared with WEAVERs to read individually at their own pace.
	Reflections	Sharing reflections and planning for upcoming session (e.g., it was suggested that ZK and PC bring clarity and structure to the design workshops, and that use of an interactive online platform may enhance collaboration).
In between: ZK and PC created tentative priorities for Design Workshop #1. This included core components of peer navigation programs (as synthesised by ZK and PC, see components labelled a–e below) and instructions for accessing and using Canva, the platform to be incorporated into design activities, as suggested, to aid collaboration.		
Design workshop #1 2 h; *n* = 7	Establishing group agreements	Reviewed mindsets towards co‐design to inspire group agreements through whole group discussion. Members added agreements to Canva board.
	Reviewing peer navigation program components (a–e)	Whole group discussion on appropriateness of these components to guide workshops.
	(a) One‐on‐one relational care from peer navigator	Smaller group (3–4 per group) brainstorming exercises using Canva, reflections shared among whole group.
	Program name	Initial brainstorming of most appropriate name for peer navigation program.
	Reflections	Closing reflections shared among whole group. Session ends 10 min early so members can also provide further reflections via online form.
In between: ZK and PC sent notes from Design Workshop #1 and priorities for Design Workshop #2. Notes included a synthesis of group agreements, responsibilities of peer navigator within relational care component and initial thoughts on program name.		
Design workshop #2 2 h; *n* = 9	Revisiting group agreements	Discussed group agreements from previous session and members absent from last workshop added ideas.
	(b) Engaging in collaborative care	Smaller group (4–5 per group) brainstorming exercises using Canva. Followed by reflections among whole group.
	(c) Building and sustaining community networks and partnerships (i.e., for peer navigators to provide warm referrals to community services)	Smaller group (4–5 per group) brainstorming exercises using Canva. Followed by reflections among whole group.
	Program name	Members added ideas for program name to a table on Canva, and then all members indicated their preferred name(s).
	Reflections	Closing reflections shared among whole group. Session ends 10 min early so members can also provide further reflections via online form.
In between: ZK and PC sent notes from Design Workshop #2 and priorities for Design Workshop #3. Notes included a synthesis of new group agreements, responsibilities of peer navigator within collaborative care and community partnerships components, and program name preferences.		
Design workshop #3 2 h; *n* = 9	(a–c) Clarifying responsibilities of peer navigator	Smaller group (4–5 per group) exercise. Groups used Canva to sort previously discussed peer navigator responsibilities (components a–c) by what responsibilities are within and outside the scope of peer navigator role. Groups also sorted responsibilities for family doctors and staff. The whole group then reconciled differences between how the two groups sorted responsibilities.
	(d) Training and support	Smaller group (4–5 per group) brainstorming exercises using Canva (one group discussed training, second group discussed support). Followed by reflections among whole group.
	(e) Tracking program progress & impact	Members asked to individually reflect using Canva on potential program benefits and what needs to be measured to understand program impact.
	Reflections	Closing reflections shared among whole group. Session ends 10 min early so members can also provide further reflections via online form.
In between: ZK and PC sent notes from Design Workshop #3 and priorities for Design Workshop #4. Notes included a synthesis of decided responsibilities, training, support, and program impact. ZK and PC also synthesised all group discussions from previous workshops into a summary, including a draft figure demonstrating the process of peer navigation based on the responsibilities designed during Workshop #3. ZK sourced key quotes from transcripts of previous workshops, which were included in the summary for members to review.		
Design workshop #4 2 h; *n* = 9	Reflecting on designed content and process	In three blocks, members were given time to individually read sections of the summary and provide individual written reflections on the wording, content and workshop process. One member elected to give her reflections verbally, which were transcribed by ZK. Members came back together and reflected as a group on their experiences in the workshops.
After: ZK and PC synthesised reflections from all group members on the summary and compiled as an internal report. ZK and PC subsequently synthesised discussions into five core components, which were reviewed by Working Group members during writing of manuscript.		

#### 2.5.2 Design Workshops

The influence that the Working Group could have on the peer navigation model for the forthcoming intervention was clearly defined. From the outset, we agreed that our outputs would inform the intervention and could be adapted according to feasibility, resourcing, and the context of different sites.

Four 2‐h design workshops with a 5–10‐min break, were held over Zoom between February and April 2025. Toward the end of each workshop, we spent time reflecting with the opportunity to provide additional reflections via an online survey, which could be completed anonymously (Table 1). With consent from Working Group members, workshops were audio‐recorded. The workshops were transcribed using Zoom, de‐identified, and checked by ZK to ensure accuracy. Members were compensated for their time ($50/hour) apart from ZK, PC, and RF who were participating in the context of their employment.

#### 2.5.3 Trauma‐and‐Violence‐Informed Approach to Design Process

We adopted a trauma‐and violence‐informed approach to our design process. Trauma‐and‐violence‐informed care is a framework for practice to support victim‐survivors that focuses on linking individual experiences of trauma to broader life conditions, accounting for “intersecting impacts of systemic and interpersonal violence” [46, 47 (p. 235)]. To facilitate collective care, we set group agreements [48], and established practices that helped us to embed principles of trauma‐and‐violence‐informed care into our processes (Table 2).

**Table 2:** Operationalising trauma‐and‐violence‐informed care in the design process.

Principles of trauma‐and‐violence‐informed care [46]	Operationalisation of principles in design process
1. Understand trauma and violence (including structural and systemic violence) and their impacts on the lives and behaviours of people, including those providing care.	– WEAVERs informed ZK and PC of accessibility requirements to facilitate planning of workshops and activities to be accessible to all members.
	– Explicitly discussing trauma‐and‐violence‐informed care in Design Workshop #1 to highlight impact of structural violence in program design.
	– Designated breaks during all workshops and emphasising members can take other breaks or turn camera off when needed.
2. Create emotionally, culturally, and physically safe environments for service users and providers.	– Prior to workshops, all members were asked to reflect on questions related to readiness to engage in co‐design, including considerations about safety, boundaries, and reasoning for engaging in the work [40].
	– Emphasis on how responses would not be associated with any identifying information (unless members would like to be identified) to help members feel comfortable voicing opinions.
	– Opportunity for anonymous reflections after each workshop and external contact available for WEAVERs to discuss any issues.
	– Established group agreements in early sessions, drawing on K.A. McKercher's six mindsets towards co‐design (e.g., elevating lived experience, practising curiosity, etc.) [48] Examples of our agreements included: recognising this process takes time; caring for each other; being realistic about what the Working Group can accomplish and influence; and being willing to explore and innovate.
3. Foster opportunities for choice, collaboration, and connection.	– Informal session with WEAVERs, ZK and PC with primary goal of getting to know each other and sharing information (session not recorded).
	– Transparency regarding impact (including limitations) of the Working Group's discussions on the forthcoming intervention
	– Transparency in reporting of methodology, including how decisions were made.
	– Emphasis on deciding what questions need answering as a group, rather than researchers setting pre‐determined priorities.
	– Smaller group discussions throughout workshops to allow all members to have the space to share ideas.
	– Discussion of how the Working Group will make decisions together, particularly in areas of disagreement.
	– Discussed outputs of project and preferences for Working Group members’ contributions to outputs.
4. Provide strengths‐based and capacity‐building ways to support service users.	– Changes to research process in response to members' preferences, for example, enabling members choice in how they were identified or not (i.e., use of actual name or pseudonym).
	– Emphasis on being mindful of how sharing personal experiences may impact self and others.
	– Drawing on specific expertise and strengths of group members.
	– After informal session, members were asked if there were any skills they wished to build throughout the process.
	– Members compensated for their time (unless participating in the context of their employment).

### 2.6 Synthesis and Refinement of Design

**Figure 1:**
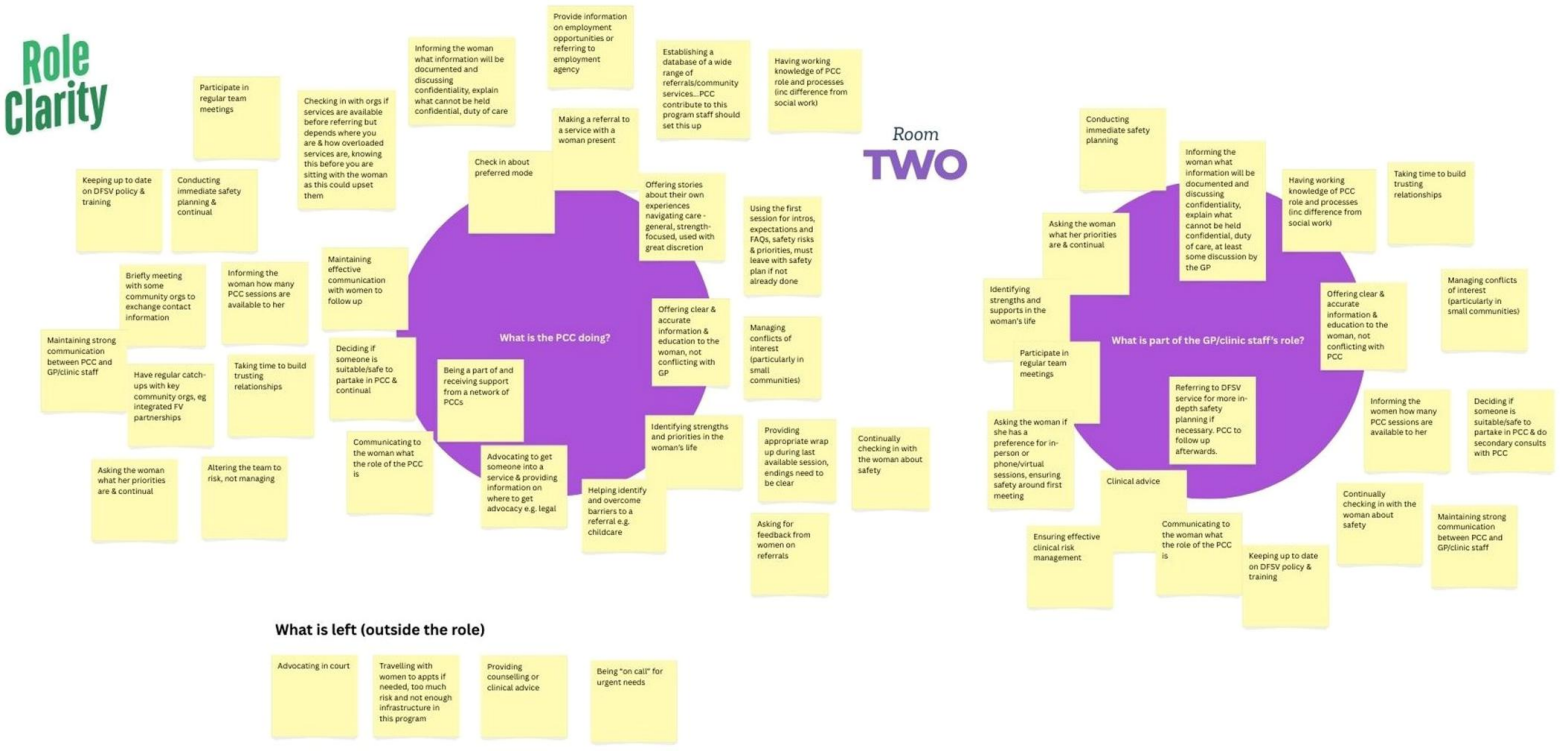
Example of design activity from canva board. This represents an example activity from one of the breakout room (small group) activities. This example does not reflect consensus made as a Working Group. PCC (“peer care coordinator”) was a name used for “peer navigator” throughout some workshops. We opt to use “peer navigator” in this paper as “coordinator” was often confused with a supervisory role. GP = general practitioner (family doctors).

We synthesised the information generated through the workshops using a design‐focused approach to construct the core components. This meant that rather than analysing transcripts or other activities as ‘data’, we used our time during workshops to design components as a group. This approach helped to generate and build on ideas among members. Outputs were generated from the tools used in the workshops (Figure 1) plus the notes and transcripts from activities and reflections. After each workshop, ZK and PC synthesised discussions into summaries that were shared with members via email.

The design was iterated and presented in a summary document that all members reviewed and provided additional contributions to in the final workshop (Table 1). ZK and PC integrated these contributions to refine the core components, which are presented along with representative quotes from workshops and reflections. Members affirmed use of quotes and edited quotes for clarity. Members chose a pseudonym for quotes or indicated they would like to be identified.

## 3 Results

The findings presented highlight our Working Group's five core components of a DFSV peer navigation program in primary care settings (Table 3). We offer these core components as general principles that those overseeing program implementation can adapt to specific contexts. Finally, we present reflections on the participatory design process.

**Table 3:** Core components of DFSV peer navigation proposed by working group.

Core program component	Description
Walking Alongside: Peer‐Informed Relational Care	Peer navigators can offer crucial emotional support based on shared lived experience, with an emphasis on doing with and for victim‐survivors. Through a foundation of relational safety, victim‐survivors can identify what matters most to themselves, and peer navigators can provide support to work towards victim‐survivors' priorities.
Peer Navigation as Practical Support	Peer navigators can work collaboratively with victim‐survivors to identify strengths, meet practical needs, and overcome barriers to accessing care and other forms of support (e.g. financial barriers, discrimination, transportation, etc.)
Safe and Ethical Practice in Peer Navigation	Highlighted as particularly critical by lived experience experts in the Working Group, this component emphasises how safety must be considered in program design (including related to informed consent, confidentiality, and documentation), especially given that peer navigation is new in DFSV responses.
Role Clarity and Boundary‐Setting	Peer navigators must be supported to maintain a clear scope of their role and to communicate their scope transparently to victim‐survivors. Adequate organisational supports (see more in following component) can support peer navigators in upholding boundaries for sustainable practice, as well as ensuring other staff members (e.g. clinicians) understand the role of peer navigators.
Organisational Supports for Peer Navigation Practice	Strong organisational structures are needed to support peer navigators. These include adequate training of peer navigators and other staff; well‐coordinated supervisory supports; and creating a culture of feedback.

### 3.1 Walking Alongside: Peer‐Informed Relational Care

The Working Group identified a central component of the peer navigator's role was drawing on their lived experience of DFSV, as well as experiences in navigating services, and applying this as lived expertise in purposeful ways to connect, validate and support. The Working Group felt victim‐survivors are likely to be more comfortable discussing experiences with a peer who understands, which was conceptualised as walking alongside victim‐survivors, offering emotional support, and relational care. Thereby, in this peer work context, ‘navigation’ is relational and responsive, grounded in what matters most to the person, so that they can problem solve collaboratively to connect victim‐survivors with support at their own pace and readiness.

[The peer navigator] is an informed sounding board, a guide, a coach, who understands the landscape through their experience.

– Liz, WEAVER

This is what makes this program special, it is about going at her pace

– Katie, WEAVER

This approach of walking alongside was considered vital to building relational safety, which for victim‐survivors who have experienced harm in the context of their relationships, can be foundational for building trust in other areas of their lives, including with services, practitioners, in relationships, and with themselves.

Some members of the Working Group were optimistic about the opportunity for peer navigation to fill crucial gaps in emotional and practical support for victim‐survivors, particularly in terms of the shortcomings and/or constraints faced by doctors.

Taking the time to build the trust and rapport, and priority setting are the most important aspects to me. Often the other roles do not have the time or capacity to develop the trust and rapport needed for the [victim‐survivor] to share their priorities with them.

– Renee, Health Practitioner

At the same time, we were cognisant that peer navigators, similar to DFSV practitioners and doctors, will encounter challenges arising from systemic inequities and the complexity of an often‐under‐resourced system. In acknowledging these challenges, we were pragmatic about the potential for peer navigators to provide survivor‐centred support within a multidisciplinary team‐based approach.

It could create a sense of a team of support for the woman, but also for the [family doctor] who can sometimes feel isolated in this work and who might otherwise feel like they carry risk alone

*–* Sally, Health Practitioner

This project can save and transform lives. If my [family doctor] had been able to do this over 17 years, then my child and I would be less damaged.

– Katie, WEAVER

### 3.2 Peer Navigation as Practical Support

Consistent with the ‘walking alongside’ approach, some Working Group members asserted that the navigation component offers victim‐survivors practical guidance to access care in a collaborative rather than directive manner. In practice, this means working collaboratively to identify strengths, goals and to make care pathways more accessible and less complex.

It's not someone else saying, ‘First we're going here, here and here’ and you feel like someone else is pulling. But the victim‐survivor can say, ‘Actually, could we go there first?’ So, you're guiding your own journey as the individual instead of someone else telling you yet again.

– Liz, WEAVER

Peer navigators may work with victim‐survivors to identify barriers, such as financial constraints, transport, discrimination, accessibility or other health concerns, and explore practical strategies to overcome them.

I think [collaborative problem solving] is what a lot of survivors are looking for and cannot find so [they] often feel very lonely during the journey.

– Renee, Health Practitioner

There was consensus that conversational and simple visual tools (Supporting Information S1: File), may help victim‐survivors recognise strengths, protective factors, and challenges they wish to focus on.

So, kind of maybe having clear information of even just branches of different support that are potentially available. So, ‘Let's look at your psychological support. Let's look at your financial support. These are the type of things that we can offer you’.

– Heshani, WEAVER

We identified that a key feature of navigation is the use of warm referrals, which extend beyond simply providing contact details for services. Warm referrals may include contacting services on their behalf (with consent), arranging introductions, helping prepare for appointments, and checking in afterwards to ensure that connections were helpful. These relational and hands‐on practices reflect peer work values and the peer navigator's role in walking alongside, rather than being overly directive. While this does not overcome issues of access, it may help alleviate the distress and frustration that victim‐survivors experience in trying to connect with appropriate and available services.

We identified that peer navigators' capacity to connect victim‐survivors with community services can be strengthened through partnerships with local organisations, particularly where referral needs may be urgent. Supervisors (Section 3.5) can support peer navigators to build and sustain these relationships, for example, by developing and maintaining a database with a range of community resources.

I definitely think the centralised [database] with the ability to identify the specifications, say particular regional areas, that it only caters to a service or is able to be identified in the database. But yeah, [the peer navigator] being able to add in [to the database] but not necessarily take control of the whole database would be good.

– Heshani, WEAVER

This includes culturally safe referral options for victim‐survivors from First Nations communities, culturally and linguistically diverse communities, people with disabilities, older people, and LGBTQ+ communities, as well as children and young people.

There was consensus that employing peer navigators from diverse cultural and social backgrounds can create more opportunities for connection and improve access to appropriate supports. When making referrals, it is also important to consider the need for translation services and translated materials to ensure accessibility. Members highlighted that offering flexible options for engagement, including in‐person, phone, and online modes, may also help to promote engagement particularly in rural communities. It is important to emphasise victim‐survivor agency in choosing referral pathways. Therefore, a challenge peer navigators may face, especially in smaller and regional or remote communities, is that there will be very few (if any) referral services or existing services may not be safe or appropriate for victim‐survivors. We discussed how community networks and adequate supervision can assist peer navigators to address challenges in linking victim‐survivors with suitable supports, such as limited referral options in smaller communities and being asked to refer to services that the peer navigator has had previous negative experiences with.

There may be biases held by the peer navigator due to their own personal experiences, which could cause tension when attempting to recommend such a service someone. This could be a particularly tricky when there are limited services for a particular population such as the LGBTQI+ community or even the South Asian community.

*–* Heshani, WEAVER

### 3.3 Safe and Ethical Practice in Peer Navigation

The Working Group emphasised that safe and ethical practice is foundational to peer navigation and presents some unique challenges when working with victim‐survivors presenting to primary care clinics. We identified ethical considerations, particularly around informed consent, confidentiality and documentation that those responsible for overseeing the implementation of peer navigation programs must pay careful attention to ensure that care is delivered responsibly and without harm to victim‐survivors or peer navigators.

Given the context of DFSV, safety was crucial for such a program. The Working Group discussed complexities in monitoring safety for peer navigators and victim‐survivors. There was consensus that those referred to peer navigators should not be experiencing high levels of harm from perpetrators; noting however, that safety may fluctuate and can escalate on short notice.

But I think it's not just the risk to the peer worker. It's the risk from the perpetrator, their supporters, any persons in the practice who are hostile to that role as well or from people in community…and I feel that risk evolves over time as well. It's really dynamic and could escalate quite quickly. So, it could be very, very dicey sometimes.

– Teresa, WEAVER

While peer navigators have an important role in checking in on safety, managing safety or undertaking complex safety planning falls beyond the scope of the role. Instead, we discussed that if peer navigators identify safety concerns or changes in safety, they are expected to seek guidance from their supervisor.

In considering the setting of primary care, several Working Group members expressed distrust in family doctors' capacities to support the intervention, particularly in terms of family doctors' abilities to assess safety and provide trauma‐informed care. One WEAVER noted:

My concern is around any [DFSV] assessment that's done by a [family doctor]. Even with training, I really don't feel like they're sufficiently skilled in that role to do that at all. It must be done by a [DFSV] service trained in [DFSV] risk assessment. I really think that that's a missing piece in all of this.

– Teresa, WEAVER

For this reason, Working Group members expressed a preference for peer navigators to be employed by, or co‐located within, DFSV services rather than primary care clinics. The rationale was that DFSV services can provide DFSV‐focused supervision and support for peer navigators, including access to specialist safety planning and referrals within the service.

There was particularly strong emphasis on ensuring that peer navigators and doctors communicate transparently about confidentiality and its limits. Members highlighted is the importance of establishing clear processes for documenting sensitive information for peer navigators and doctors.

I think it's really important that the [family doctor] has a specific training on how to document family violence in a file.

– Sally, Health Practitioner

We considered that in Australia, current legislation enables documented information to be subpoenaed by courts (in family, criminal and civil proceedings). Given this, peer navigators and doctors must be transparent and obtain informed consent so that victim‐survivors understand how their information will be recorded, stored, and accessed, including the potential for it to be subpoenaed.

### 3.4 Role Clarity and Boundary‐Setting

In response to the complexity of safety considerations, we identified the importance of establishing a clear scope for the peer navigator role that can be understood by peer navigators themselves, those working alongside them, and victim‐survivors whom they support. While peer navigation is flexible, it is critical for peer navigators to establish and maintain the boundaries of their role and are transparent in what they can and cannot assist with. Thus, in defining the scope of role, the Working Group distinguished key responsibilities that differ between peer navigators and other team members, such as doctors, social workers and other specialised DFSV practitioners.

A key question to clarify is what the difference between the social worker and the peer navigator is, as there are similarities.

– Heshani, WEAVER

Distinguishing between the different roles enabled us to delineate responsibilities that are outside the scope of the peer navigator role in this context. Importantly, managing clinical safety, providing counselling, and being ‘on call’ are beyond the scope of the peer navigator's responsibilities.

Given that peer work roles are relatively new in primary care contexts, particularly in the context of DFSV, there was consensus that family doctors and other practitioners must have a clear understanding of and respect for peer navigators' roles and responsibilities. Clarity among the broader team is essential to supporting peer navigators to maintain appropriate boundaries. We identified that while peer navigators can have a strong sense of their own boundaries, these can be difficult to uphold if the scope of their role is not well understood or respected by doctors, especially those who have not previously worked with peer workers.

[Clinic staff] need to have very clear understanding of the role and how it fits with the clinic, within the team and with the patients. If they have clarity around this, they will be instrumental in helping the role to be effective.

– Sally, Health Practitioner

Equally important, and as part of informed consent, is clear communication of the peer navigator's role to victim‐survivors by both peer navigators and the referring doctors. It was suggested that communication materials be developed to support clear and consistent explanations of this information that outlines how the program will run, the number of sessions, and limits to the program.

As peer workers, the peer navigators may disclose aspects of their own experiences when appropriate and helpful. While this can build connection, maintaining clear boundaries is essential to the integrity of peer work. In exploring this, we affirmed that self‐disclosure should be managed thoughtfully and in a way that centres the victim‐survivor's experience rather than the peer navigator. Therefore, it is important for both peer navigators and victim‐survivors, that peer navigators are mindful of when and how to share their stories.

[Not] oversharing and keeping our own stories brief in a way that is relevant to the victim‐survivors individual experiences can provide comfort and consolidation by building a ‘bridge’ of understanding and reducing the victim‐survivors isolation. Prioritising listening to validate their experiences is important before sharing our own story as well as pivoting the conversation back to their needs.

– Shiana, WEAVER

Ongoing training and supervision were emphasised as crucial in supporting peer navigators to apply their lived expertise safely, effectively, and sustainably (Section 3.5). We also recognised that in recruiting peer navigators, acknowledgement and reflection on where they are in their own journey toward recovery is crucial to ensure readiness.

### 3.5 Organisational Supports for Peer Navigation Practice

The Working Group discussed the organisational structures that would be conducive to fostering safe, ethical, and sustainable peer navigation practice. Ongoing training, supervision and reflective practice were identified as critical supports to help peer navigators manage the complexities of maintaining boundaries, upholding confidentiality, and working safely within their scope.

Equally important are processes that embed peer navigators within primary care settings in ways that promote shared understanding and collaboration. As mentioned previously, family doctors and DFSV practitioners must be well supported to understand the peer navigator role and to engage in meaningful, coordinated practice that ensures peer navigators are integrated effectively within the care team.

A peer navigator may have excellent skills, knowledge, and the ability to perform their role effectively. However, if the clinic cannot adequately support that role or the work involved, the effectiveness of the position can quickly unravel.

– Sally, Health Practitioner

Training and communication processes were identified as playing a vital role in building this shared understanding. The Working Group felt strongly that doctors, peer navigators, and clinic staff should undergo training in trauma‐and‐violence‐informed care and training to prepare for embedding peer work. Regular communication channels such as care coordination meetings and participation in a community of practice can promote reflective practice and mutual learning.

We identified that peer navigators may face multiple, intersecting challenges in their work that increase the risk of re‐experiencing trauma, vicarious trauma and burnout. To mitigate this, the Working Group identified the need for flexible, tailored, and well‐coordinated supports, particularly supervisory supports. We identified two key supervisory roles: (1) Program Manager who provides central coordination and line management for peer navigators; and (2) Peer Work Practice Supervisor who provides specialised guidance on complex cases and facilitates reflective practice, and professional development to support trauma‐and‐violence‐informed care. The latter role is akin to professional supervision that is typically offered in mental health and peer work contexts, and which focuses on reviewing cases, building reflective capacities, understanding boundaries, and applying ethical practice.

There was consensus that peer navigators would also benefit from a community of practice in which regular meetings with other peer navigators can provide ongoing opportunities for shared learning, problem‐solving, and mutual support.

A third type of supervision being peer supervision, so that they would have an opportunity to work with the other [peer navigators] who are doing similar jobs and so that they can, I guess, discuss some of the challenges and how they've overcome them and share ideas and feel like they have a team of other workers doing similar work.

– Sally, Health Practitioner

Additional counselling, workshops or wellbeing support can also be implemented, such as support for reactivated trauma and burnout. Flexible approaches to support and supervision were also discussed by Working Group members to ensure that peer navigators, who bring diverse educational and professional experiences, are equipped to fulfil their roles safely and effectively.

Finally, we recognised that peer navigators can foster a culture of feedback by inviting victim‐survivors to share their experiences of accessing community resources, strengthening the responsiveness and quality of support over time.

We talked about the importance of continual feedback and that that was really important, and perhaps having non‐verbal feedback as well

– Shiana, WEAVER

Similarly, it was felt that doctors can also seek feedback on victim‐survivors’ experiences with peer navigators. Gathering feedback needs to be carefully managed at the organisational level to ensure that feedback is shared in constructive and safe ways. We determined that these processes can be facilitated in supervision settings and informed consent needs to be carefully obtained.

### 3.6 Reflections on Design Workshops

Members provided reflections on the participatory design process and the work produced. Members were proud of how much the Working Group accomplished through the workshops, emphasising that the Working Group promoted “psychological safety” and that it was a “respectful space”. Underpinning this were clear design priorities for each workshop and group agreements which provided a foundational structure to guide the Working Group in meaningfully applying their lived expertise.

[Using] our experience through expertise and ideas rather than very detailed individual experience sharing, which is not always helpful in projects (can be distressing) and can derail the process and ability to input.

– Liz, WEAVER

The flexibility for design priorities to evolve, and for members to shape the direction of the workshops, was highly valued as a mechanism to facilitate power sharing.

It was a great example of power sharing, we were assumed as coming with our own autonomy, strengths and resources. The mutual sharing of power was healing. The project was purposeful. We could see the outcomes it would lead to for clients and workers. It was true co‐production at its best.

– Katie, WEAVER

The process has been a true codesign and I feel very heard and that I have been able to have influence through the sessions. This has made me feel empowered and builds my confidence also.

– Liz, WEAVER

Taking time to consider how potential safety concerns could impact the program was seen as an important part of the design process, with one member indicating she was most proud of:

Making a difference and highlighting risks and opportunities for better programs and policies.

– Teresa, WEAVER

Working Group members highlighted the use of interactive design tools and activities, including small group discussions and online platforms as useful in facilitating engagement in multiple ways. While this approach sought to offer multiple ways of members engaging in tasks, some online tools were not accessible to all members. Allowing for more time and guidance to support access to these is an important consideration for improving the experience for all members.

The final workshop focused on refining the design and reflecting on work completed, rather than introducing any new material, which Working Group members reported appreciating. This also included reviewing quotes for inclusion in reports and publications arising from the project, which served as tangible evidence of everyone's contributions to the outputs. While the work we set out to complete was undertaken feasibly within the four workshops, the Working Group expressed interest in continuing to collaborate by contributing to project outputs.

## 4 Discussion

Through a participatory design approach, this study responds to gaps in knowledge to guide the implementation of peer navigation for victim‐survivors of DFSV in primary care settings. Our Working Group consisting of women with lived experience of DFSV, health practitioners, and researchers highlighted how peer navigators can provide crucial relational and practical support through ‘walking alongside’ victim‐survivors as they navigate complex DFSV systems. Our findings point to contextual and pragmatic implementation considerations at the organisational level that enable safe and ethical peer work practice. Among these considerations, key priorities include clearly establishing peer navigator scope of practice, as well as ensuring organisational understanding of peer work principles and ways of working, which are distinct from clinical roles. Importantly, the effectiveness and sustainability of peer navigation in primary care is contingent on supporting the safety and wellbeing of peer navigators. Foundational to this are mechanisms and resourcing of comprehensive recruitment, training, supervision and reflective practice, which must be embedded within broader trauma‐and‐violence‐informed systems at an organisational level.

The capacity for peer work in the DFSV field to provide relational care that centres victim‐survivor agency has been widely recognised [8, 16, 23, 25]. In the mental health field, relational care has similarly been described as a central to peer work values and practices [26, 28, 49]. Our findings reinforce these perspectives and the lived expertise of our Working Group highlighted victim‐survivors’ desire to have someone ‘walk alongside’ them and take the time to ask for their own priorities for support, tasks that clinicians often do not have time for [6, 50, 51, 52, 53]. This idea of ‘walking alongside’ can also be conceptualised as ‘sitting’ or ‘being’ alongside to promote more inclusive language [54]. We also highlight the importance of providing opportunities for victim‐survivors to connect with peer navigators whose shared lived experience extends beyond DFSV and may also include other aspects of their identities, circumstances, and experiences of structural exclusion or marginalisation [35, 55, 56]. This is central to the provision of trauma‐and‐violence‐informed care, particularly with regard to enabling inclusive and culturally safe care [12, 14, 46, 47].

The DFSV workforce are one of the most at‐risk groups for occupational stress including secondary stress [57, 58, 59, 60, 61, 62]. The risks for stress may be compounded for peer navigators whereby their own experiences may be mirrored in those of the victim‐survivors they work with [23, 58, 63], a concern raised by our Working Group. At the same time, peer work can also be healing, with benefits such as sustaining a sense of purpose, recontextualising experiences, and facilitating posttraumatic growth [23, 58, 63, 64]. The importance of strong organisational and supervisory structures to support DFSV peer navigators is central to our design. The necessity of comprehensive organisational supports is echoed in the broader literature, particularly in mental health, as being fundamental to supporting peer navigators in managing boundaries and preventing burnout [63, 65, 66, 67].

We emphasised that supervision for peer navigators should be undertaken by practitioners with expertise in supervising DFSV workers. These considerations were affirmed by one of the few DFSV peer navigation programs in the literature, which embedded peer navigators within a culturally specific DFSV service [35]. At the same time, access to supervisors with specialised experience in peer work is critical in supporting peer workers, and is best practice in the mental health field [67]. A scoping review of supervision in the mental health peer workforce highlighted that previous experience in a peer role and familiarity with peer work values are necessary for peer work supervisors [68]. While there is a high proportion of the DFSV workforce with lived experience [58, 61, 69, 70], designated lived experience or peer roles are still emerging in this field. Therefore, DFSV services embedding peer workers and peer navigators must contend with how to provide supervision that is grounded in peer work values and expertise while lived experience roles in the sector are still emerging.

Equally important to supporting peer navigators, is understanding how family doctors can be supported to engage with the program, particularly given the demands and constraints of primary care (e.g., brief time for appointments) [11]. Important considerations were centred around support for doctors in assessing and managing safety and ensuring informed consent, which have been identified as barriers to survivor‐centred care [11, 20]. While some members of our Working Group expressed a lack of trust in family doctors' abilities to appropriately support victim‐survivors, we also acknowledge victim‐survivors are frequently accessing health services meaning family doctors must be able to provide appropriate and trauma‐and‐violence‐informed care [8]. Establishing team‐based approaches with peer navigators and DFSV practitioners is one pathway to better support family doctors. Access to peer navigators can address time‐based barriers to support that family doctors are not often able to overcome, and collaboration with DFSV practitioners can allow for doctors to improve self‐efficacy and increase access to specialist support [53].

As research on DFSV peer work is still emerging [23], our Working Group spent time exploring how peer navigators would attend to potential safety and ethical considerations including presence of perpetrators, conflicts of interest especially in smaller communities, and documentation of information, particularly when files may be subpoenaed and used in legal proceedings. The focus on safety in the design of the program stemmed from, and was enriched by, the lived expertise of our Working Group. It also parallels how safety—and by extension, risk—are conceptualised in medical and DFSV specialist settings. However, we acknowledge that discussions of ‘risk’ that arose in our Working Group are often conceptualised in different ways in peer work practice, particularly in mental health [24, 71]. Peer work seeks to reframe narratives of risk, whereby rather than managing all possibilities of risk, there is a focus on “having the difficult and honest conversations with peers and trusting them to manage themselves” [24 (pNP14057), 71]. Similarly, to the mental health sector, there are tensions between existing clinical and DFSV practice surrounding ‘risk’ and the practice of peer work in this context of DFSV [71]. Consequently, these services must consider how peer work values and practice can sit alongside existing clinical care and professional practice where safety assessment and risk management are often central to working with victim‐survivors [24]. As evidence grows, future work is needed to support meaningful integration of peer work values and primary care practice to enable a collaborative approach to survivor‐centred care.

### 4.1 Strengths and Limitations

Central to the ethos of a peer navigation program is the focus on lived experience as expertise, thus, the choice of participatory methods was intentional and a strength of this study. We designed the program's core components in response to the lived expertise of our members, enabling the design to be attentive to the values of peer work and responding to challenges peer navigators may face. Women with lived experience of DFSV were involved in study conceptualisation, design activities and synthesis, and manuscript authorship, embedding lived expertise throughout the study. Further, we brought together a group where there were some existing relationships and where all members had some experience in co‐production and/or program design. This supported us in navigating challenges in the process that were complex or where consensus was harder to reach.

There were also some constraints that shaped our approach, most notably a pre‐determined timeframe in which to complete the design (approximately 6 months). To ensure momentum, we used multiple formats for communication both during and between workshops, including small and large group discussions, online tools, and options for anonymous feedback. Overall, we took a pragmatic approach to determining our priorities and what we could accomplish as a group. This necessitated two members (ZK, PC) synthesising and consolidating the design between workshops and sharing this with the broader group and we acknowledge the shift in power sharing through this part of the design process as a limitation (Table 1). In light of these constraints, our Working Group aimed to be transparent about the design scope, the parameters of influence within and outside of workshops, including through the writing of draft reports and co‐authoring of the manuscript.

Further limitations included the lack of representation of family doctors in the Working Group. The absence of family doctors did support lived experience experts in representing the majority voice of the Working Group and empowering members to discuss concerns about primary care. At the same time, future work is needed for implementing organisations to build on these proposed core components and operationalise processes such as screening and referral processes in consultation with family doctors and DFSV practitioners. Group members from the WEAVERs brought a deep amount of lived expertise to the study. Simultaneously, we acknowledge the limitations of working with experts from one organisation, particularly in relation to tensions surrounding representation, as previously reported by the WEAVERs [72, 73]. Future work should continue to centre to the lived expertise of victim‐survivors in the design of the forthcoming intervention and peer navigation programs more broadly as well as to tailor the program design for varying contexts and populations.

## 5 Conclusions

Through the lived and professional expertise of our Working Group, our findings demonstrate how the implementation of peer navigation programs for victim‐survivors can provide survivor‐centred relational and practical support. Peer navigation programs supporting victim‐survivors in primary care must emphasise safe and ethical practice with clear boundaries and scope of responsibilities for peer navigators. Essential to this are whole‐of‐clinic training, DFSV‐informed supervision, and strong organisational supports. The implementation of peer navigation programs must promote care that prioritises trusting relationships, victim‐survivor choice and agency, and pragmatic support.

## Author Contributions

**Zoe King:** conceptualisation, investigation, funding acquisition, writing – original draft, methodology, visualisation, writing – review and editing, formal analysis, project administration, resources. **Heshani Samantha De Silva:** investigation, visualisation, writing – review and editing, formal analysis. **Katie Alexander:** investigation, visualisation, writing – review and editing, formal analysis. **Liz John:** investigation, visualisation, writing – review and editing, formal analysis. **Shiana Harris:** investigation, visualisation, writing – review and editing, formal analysis. **Teresa:** investigation, visualisation, writing – review and editing, formal analysis. **Renee Fiolet:** investigation, visualisation, writing – review and editing, formal analysis, funding acquisition. **Sally Connell:** investigation, writing – review and editing, visualisation, formal analysis. **Sally Nathan:** conceptualisation, writing – review and editing, methodology, supervision. **Ruth Wells:** conceptualisation, methodology, writing – review and editing, supervision. **Kelsey Hegarty:** conceptualisation, funding acquisition, writing – review and editing, methodology, supervision. **Patricia Cullen:** conceptualisation, investigation, funding acquisition, writing – original draft, methodology, writing – review and editing, visualisation, formal analysis, project administration, supervision, resources.

## Ethics Statement

This study was approved by the University of New South Wales Sydney Human Research Ethics Committee (iRECS7467).

## Conflicts of Interest

The authors declare no conflicts of interest.

## Supporting information


Supporting File

## Data Availability

The data are not publicly available due to privacy or ethical restrictions.
