# Supplementary material for: Designing a Peer Navigation Program Alongside Women With Lived Experience of Domestic, Family, and Sexual Violence
Source: Health Expect. 2026 Sep 22;29(5):e70877. doi: 10.1111/hex.70877 (PMC13598425; doi:10.1111/hex.70877)
Supplement: Supplementary file 1 [file HEX-29-e70877-s001.docx]

Supplementary File. Example Visual Tool for Peer Navigators to Assist Victim-Survivors in Identifying Strengths and Priorities for Change

Note: Made using Canva.
